# Bacterial Composition Across Bat Species: A Human Health Perspective

**DOI:** 10.3390/ani15213126

**Published:** 2025-10-28

**Authors:** Julio David Soto-López, Pedro Fernández-Soto, Antonio Muro

**Affiliations:** 1 Infectious and Tropical Diseases Research Group (e-INTRO), Biomedical Research Institute of Salamanca, Research Centre for Tropical Diseases (IBSAL-CIETUS), Faculty of Pharmacy, University of Salamanca, 37008 Salamanca, Spain; jdjuliosoto@usal.es (J.D.S.-L.); ama@usal.es (A.M.); 2 Salamanca Biomedical Research Institute, University of Salamanca, Miguel de Unamuno Campus S/N, 37007 Salamanca, Spain

**Keywords:** bats, bacteria, metagenomic, microbial, pathogen

## Abstract

**Simple Summary:**

Bats are one of the most diverse groups of mammals, with almost 1400 species found across nearly every part of the world. They play important roles in nature by helping pollinate plants, spread seeds, and control insect populations. However, bats can also carry a wide variety of microbes, some of which may cause disease in humans and animals. Recent outbreaks of illnesses that started in animals have drawn attention to the study of bacteria found in bats. In this work, we reviewed published studies from the last five years that used modern genetic tools to identify bacteria living in or around bats, especially in their droppings. We found reports of thousands of bacterial species, including some that are already known to cause infections in people. While many of these microbes are harmless or only dangerous in special cases, others are considered global health priorities because they are linked to serious diseases or are resistant to treatment. Understanding which bacteria are most commonly shared among bats may help scientists develop early warning systems for future outbreaks. Our study also shows the need for clearer and more consistent reporting methods so that this information can be better used to protect both public health and animal welfare.

**Abstract:**

Bats are widely recognized as reservoirs of diverse bacterial pathogens with important implications for human health. Recent zoonotic disease outbreaks have intensified interest in bat microbiomes, with high-throughput sequencing increasingly used to assess microbial diversity. In this article, we review literature from the past five years on bacterial species associated with bats and their potential clinical relevance. Using automated searches and manual filtering, we extracted data from 47 peer-reviewed studies. Most research has focused on guano samples, though interest in skin microbiomes is rising, particularly in relation to *Pseudogymnoascus destructans*, the agent of white-nose syndrome. China leads in the number of publications, followed by the United States, and amplicon sequencing remains the predominant metagenomic method. Across studies, 4700 bacterial species were reported, including several known human pathogens capable of aerosol transmission or opportunistic infections in immunocompromised individuals. Many of these taxa are classified as global priority targets for antimicrobial drug development by the World Health Organization and the U.S. Centers for Disease Control and Prevention. Given the clinical severity of diseases linked to some species, bats should be integrated into epidemiological surveillance systems. However, the lack of standardized reporting practices significantly limits the comparability and utility of bat microbiome data for robust ecological and epidemiological analyses.

## 1. Introduction

The order *Chiroptera* (bats) is the second most diverse mammalian order, comprising nearly 1400 of the more than 6400 described mammalian species (20%) to date [1,2]. Historically, bats have been classified into two suborders based on phylogenetic analyses: *Yinpterochiroptera* and *Yangochiroptera* [3]. Their remarkable diversity is attributed to their global distribution, as they inhabit nearly all regions of the planet except polar zones, deserts, and certain remote islands [2]. Bats play crucial ecosystem roles, serving as primary pollinators for numerous plant species [4], seed dispersers, and key regulators of insect populations [5]. They exhibit a wide range of dietary specializations, including insectivores, frugivores, carnivores, omnivores, nectarivores, and sanguinivorous [6].

Recent outbreaks of zoonotic diseases have intensified scientific interest in bats. Studies on their microbiomes reveal that, unlike other mammals, the bat microbiome is shaped primarily by ecological factors [7] rather than phylogenetic relationships. It also dynamically shifts over time within gregarious colonies [8] and is influenced by the environment [9,10]. This microbial plasticity, owing to their unique ecology, positions bats as compelling models for investigating the diversity, functions, and adaptations of bacterial communities associated with them.

Various pathogens of public health importance have been detected in bats, including more than 1400 viruses [11,12], such as SARS-CoV [13], MERS-CoV [14,15], SARS-CoV-2 [16], NL63 alfacoronavirus [17], Nipah virus [18] and Ebola virus [19], as well as viruses specific to the diet of these mammals (fungi, plants, insects) [20,21]. Protozoans such as *Bartonella mayotimonensis* Lin et al., 2010 [22], *Babesia canis* (Piana & Galli-Valerio, 1895) [23] and *Polychromophilus* spp. [24] have also been described. The guano of bats inhabiting caves and buildings often contains pathogenic enteric bacteria, as well as other bacterial agents responsible for human and animal diseases. These include species from genera such as *Pasteurella*, *Salmonella*, *Shigella*, *Escherichia*, *Klebsiella*, *Proteus*, *Yersinia*, *Hafnia*, *Serratia*, *Staphylococcus*, and *Campylobacter*, among many others [25].

Since the early 2000s, the field of human microbiome research has expanded rapidly, driven by extensive studies in large populations that have deepened our understanding of microbial diversity and revealed possible connections to metabolic well-being and a range of diseases [26]. A wide range of organisms have been described in bats and other mammals through various methodologies, including serological tests, polymerase chain reaction (PCR), virus isolation and metagenomics [12,27,28,29]. However, the vast majority have been identified through high-throughput sequencing (HTS) [30,31,32]. While these technologies were established over the past decade [33], they continue to evolve and improve.

Beyond their relevance as potential reservoirs of zoonotic pathogens, the microbiota of bats also plays a pivotal role in host physiology, immune regulation, and overall welfare [27,28,29]. Ecological and environmental mechanisms are key determinants of bacterial community composition in bats. Factors such as dietary specialization, roosting behavior, social aggregation, habitat disturbance, and exposure to environmental stressors can modulate the structure and stability of their microbiomes [8,9]. Environmental contaminants, urbanization, and changes in land use may disrupt the microbial balance, potentially leading to dysbiosis and compromised immune function [10]. Likewise, physiological stress associated with torpor, reproduction, migration, or captivity can alter microbial assemblages and increase susceptibility to infection [27,28,29].

Numerous articles are published every year that use HTS to describe the microbiome of bat samples, and in this article, we provide a comprehensive narrative review of the literature from the last five years, encompassing both quantitative and qualitative studies focused on the microbial diversity of bat guano, with particular emphasis on the presence and clinical significance of bacterial species, some of which may pose potential pathogenic risks to humans.

## 2. Materials and Methods

Although this work constitutes a narrative rather than a systematic review, general quality and bias criteria were considered during the selection and interpretation of the studies. Only peer-reviewed articles providing explicit methodological descriptions and clear taxonomic identification of bacterial taxa were included. Studies lacking methodological transparency, reporting inconsistencies, or insufficient data for species-level identification were excluded. When interpreting the results, emphasis was placed on the reliability of bacterial identification and the representativeness of the sampled bat populations to minimize potential bias in data extraction and synthesis.

**Figure 1:**
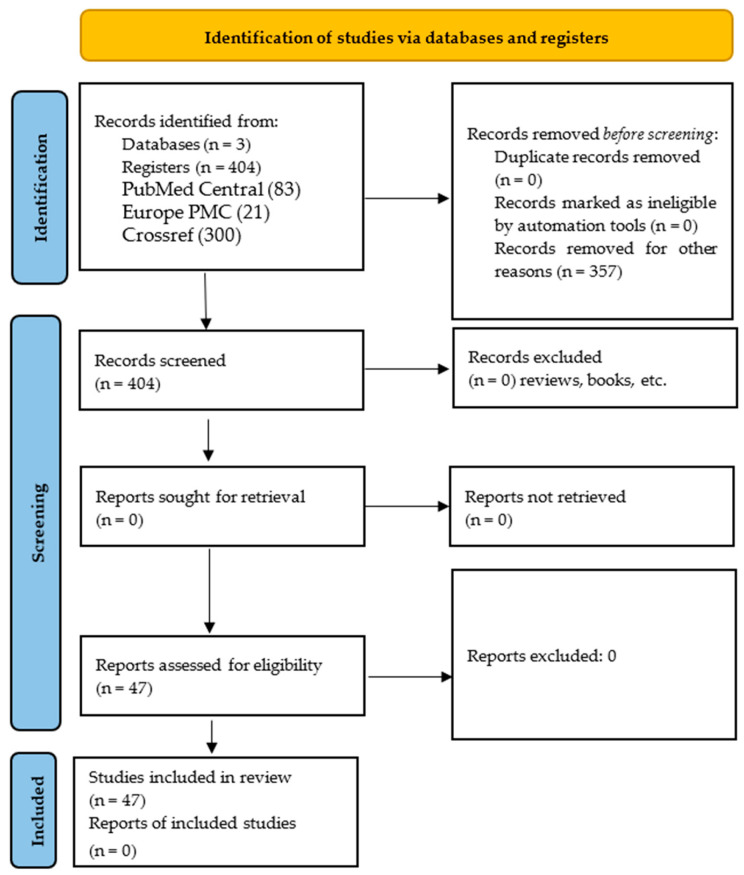
PRISMA 2020 flow chart. Only database and registry searches are indicated.

For this narrative review, we retrieved all studies focusing on bat-associated bacteria identified by metagenomics methodologies. We searched PubMed Central, Europe PMC and Crossref from 1 January 2020 until 12 May 2025. The search terms used (search string) were “Chiroptera” [MeSH Terms] OR chiroptera[TIAB] OR bat[TIAB] OR bats[TIAB]), ‘AND (“Metagenomics”[MeSH Terms] OR metagenomic[TIAB] OR metagenomics[TIAB]’, ‘OR microbiome[TIAB] OR microbiota[TIAB])’, ‘AND (“Bacteria”[MeSH Terms] OR bacteria[TIAB]))’, ‘AND (“2020/01/01”[DP]: “2025/12/31”[DP])’, ‘NOT (“Virus”[MeSH Terms])’, obtaining a total 83 hits in PubMed Central, 21 in Europe PMC and 300 in Crossref. Records were downloaded from the databases via the libraries rentrez [34], europepmc [35] and rcrossref [36] in R [37]. The inclusion and exclusion criteria were as follows: only studies dealing with bacteria isolated from bats were included, and those analyzing viruses, bacteria of other species, records without bat taxonomy, inaccessible articles, review articles, book chapters, letters, editorial material, meeting abstracts, clinical trials, meta-analyses, and systematic reviews were excluded (Figure 1).

After duplicate studies, reviews and missed hits were manually eliminated, 47 records remained (see Supplementary Table S1). All the bacterial species reported in the records were manually extracted and saved in an SQLite database via the DB Browser v 3.13.1 [38]. All records were formatted and graphed in R. The script utilized in this review can be recovered from https://github.com/jdjuliosoto/Bacterial_Composition_2020-2025 (accessed on 25 September 2025).

## 3. Results and Discussion

### 3.1. Trends in Bat Microbiome Studies

Over the past five years, research has continued to characterize the composition of bacterial communities present in bat guano (see Supplementary Table S1). Studies have focused primarily on samples obtained from fresh fecal droppings or accumulated guano deposits (28/47) in various locations, including caves, buildings, and zoological facilities. Additionally, investigations have examined the skin microbiota of bats (13/47), with particular interest increasing due to the presence of the pathogenic fungus *Pseudogymnoascus destructans* (Blehert & Gargas) Minnis & D.L. Lindner, 2013, which affects bat populations. The country with the greatest number of studies on this subject is China, with 21% (10/47) of the articles, followed by the USA with 13% (6/47), and the remaining countries with fewer than 6% (3/47) of the articles per country, a trend that has been observed for several years [39]. The predominant tool for the identification of bacteria is high-throughput 16S rRNA sequencing, with 83% (39/47) of the articles using this method, followed by cultivation methods (15%, 7/47), high-throughput shotgun sequencing (5/47) and PCR (4%, 2/46).

### 3.2. Bacteria Associated with Bats

**Figure 2:**
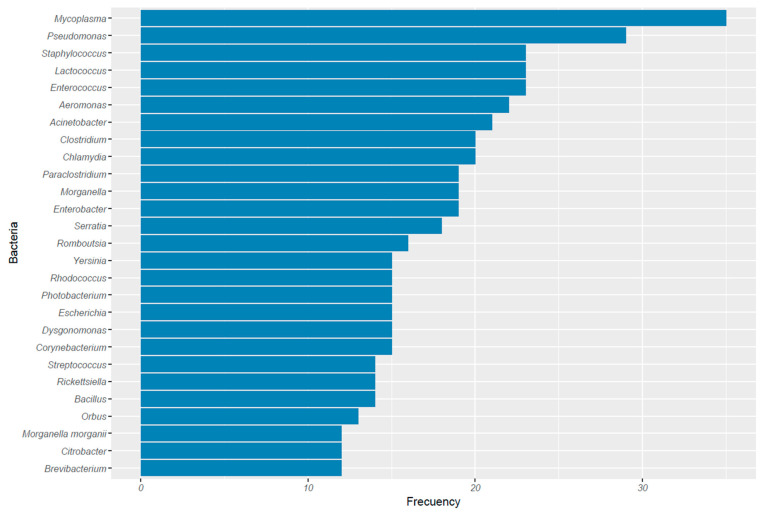
More abundant bacteria registered in published articles between 2020 and 2025.

Significant bacterial diversity has been identified in guano samples, with variations observed depending on the bat species, sample types, and specific sampling sites—such as different cave locations or layers within guano deposits. The more frequently reported bacteria (Figure 2) have several common clinical and microbiological characteristics.

Many of these genera are pathogens of humans or at least opportunistic in immunocompromised individuals (e.g., *Mycoplasma*, *Staphylococcus*, *Pseudomonas*, *Acinetobacter*, *Enterobacter*, *Clostridium* and *Chlamydia*) [40]. Some of them are associated with nosocomial infections (e.g., *Enterococcus*, *Klebsiella* and *Serratia*). Some strains are even known for their ability to develop resistance to multiple antibiotics (e.g., *Pseudomonas*, *Acinetobacter*, *Staphylococcus*, *Enterobacter*, and *Escherichia*) [41]. Most of these genera are commonly found in the digestive microbiome of mammals or in wild environments (*Escherichia*, *Enterococcus*, *Staphylococcus*, *Streptococcus*, *Corynebacterium* and *Bacillus*) [7,42]. This reflects the importance of bats as a source of pathogenic bacteria, or at least as reservoirs.

**Figure 3:**
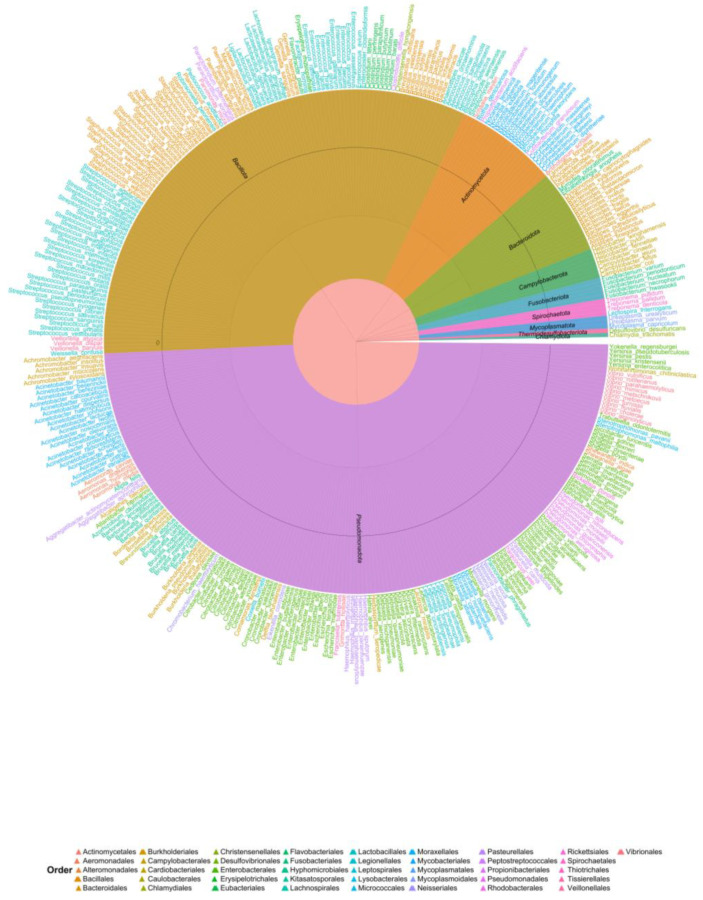
Bacteria were isolated from humans with documented pathologies at the *Bacterial and Viral Bioinformatics Resource Center (BV-BRC)* as of 19 May 2025 [43]. Each tip represents a bacterial species. Species colors correspond to bacterial orders, whereas circle colors indicate phyla.

The revised articles also show that although not all articles reported their results at the taxonomic species level, a total of 4700 different bacterial species were recovered (Supplementary Table S2). Bacteria isolated from humans with various diseases (according to the BV-BRC) (Figure 3) are also present in bats. This results in bats functioning as reservoirs of several bacteria with high impacts on human health. Among the bacteria isolated from human patients with a disease, the most common genera belong primarily to two major phyla (*Firmicutes*/*Bacillota* and *Proteobacteria*/*Pseudomonadota*). These results suggest that bats harbor bacteria ranging from mucosal commensals (*Lactobacillales*, *Corynebacteriales*) to opportunistic and classic pathogens (*Enterobacterales*, *Pseudomonadales*, *Clostridiales*). Many of the bacteria associated with a sick patient cause some type of infection (Table 1).

Dimkić et al. (2021) [25], divided, from a clinical significance, bacteria from guano into four groups, which we also found useful for use. The first group included bacteria classified as enteric foodborne or other pathogens. In this group, they include genera such as *Escherichia*, *Enterobacter*, and *Yersinia*. The second group comprises common zoonotic pathogens that include the genera *Bartonella*, *Borrelia*, *Leptospira*, *Campylobacter*, *Clostridium*, and *Bacillus*. The third group includes unusual Gram-negative bacterial pathogens with atypical cell structures, including the genera *Mycoplasma*, *Ureaplasma*, *Rickettsia*, *Anaplasma*, and *Chlamydia*. The fourth group includes extended-spectrum beta-lactamase (ESBL) and carbapenemase-producing *Enterobacteriaceae* (CPE). All the genera mentioned by these authors were also found and reported in recent articles (Figure 2, Supplementary Table S2).

Among the bacteria that are found in guano, some cause dangerous diseases whose transmission may be obscured because they can be transmitted by aerosols (“droplet nuclei” of 1–5 µm) and others that cause severe and difficult treatable diseases (Supplementary Table S5). The potential risk of spillovers of those bacteria points to the importance of the One Health perspective in the surveillance process. As previously reported by many authors [25,30,31], pathogens are common in guano residents; therefore, guano piles are potential reservoirs for the spread of zoonoses. Zoonosis events are more likely to occur in bats and humans because both often share habitats (church tower, building–dwelling, farms) [45,46], which could facilitate direct or indirect transmission (through feces, saliva, and insect vectors).

Among the vast number of bacterial species in bats, several bacteria classified as priorities in the development of drugs for their control are described by both the World Health Organization (WHO) and the Centers for Disease Control and Prevention (CDC) in their report “*Antibiotic Resistance Threats in the United States*, 2019”. We found that the revised articles indicate the presence of *Enterococcus faecium* (Orla-Jensen 1919); Schleifer & Kilpper-Bälz 1984, *Haemophilus influenzae* (Lehmann & Neumann 1896), *Helicobacter pylori* (Marshall et al., 1985) Goodwin et al., 1989, and *Mycobacterium tuberculosis* Zopf, 1883 as priorities for the WHO [41]; *Bordetella pertussis* (Bergey et al., 1923); Moreno-López 1952, *Clostridioides difficile* (Hall & O’Toole, 1935) Lawson & Rainey, 2016, and *Mycoplasma genitalium* Tully et al., 1983 as priorities for the CDC [47]; and *Escherichia coli* (Migula 1895); Castellani & Chalmers 1919, *Neisseria gonorrhoeae* (Zopf 1885) Trevisan 1885, *Pseudomonas aeruginosa* (Schroeter 1872); Migula 1900, *Staphylococcus aureus* Rosenbach 1884, and *Streptococcus pneumoniae* (Klein 1884); Chester 1901, along with the genera *Acinetobacter*, *Campylobacter*, *Enterobacter*, *Klebsiella*, *Salmonella*, and *Shigella* as priorities for both the WHO and the CDC.

Bats are widely considered ideal hosts for a large range of pathogens because of their combination of intrinsic and extrinsic traits. Among intrinsic factors, their immune response stands out as a critical determinant of their efficiency as reservoirs. Bats exhibit tolerance to viral infections, driven by genetic variations in the genes encoding receptors through which pathogens enter their bodies [48]. Together, these adaptations enable bats to harbor diverse pathogens asymptomatically [49]. Bats exhibit prolonged longevity (e.g., 41 years in *Myotis brandtii* (Eversm, 1845), which facilitates long-term persistence of pathogens within their populations [50]. Depending on the species, they can be homeothermic or heterothermic and capable of hibernating or entering torpor [51], a state where their body temperature drops drastically to match the ambient temperature of their hibernaculum (e.g., 5–10 °C), making them susceptible to infections. They rely on echolocation [52] and magnetoreception [53], employ reproductive strategies to delay fertilization, and combined with their true flight capability, establish migratory patterns [54] that promote pathogen dispersal to distant regions.

Among extrinsic factors, the large colony sizes of chiropterans—often reaching hundreds of thousands of individuals—facilitate efficient pathogen transmission between bats [48]. On the other hand, habitat loss driven by continuous urban expansion has increased roosting opportunities for bats within human communities [55]. This heightened proximity elevates stress levels in bats, leading to weakened immune systems and consequently amplifying pathogen transmission among individuals, directly impacting disease epidemiology [56]. Additionally, the use of bats as a food source on Pacific islands, parts of Southeast Asia, and Madagascar [57] underscores their importance in public health [58].

The bacteria prioritized by the WHO and CDC described in the revised articles are considered because of their mortality, health-care burden, prevalence of resistance, trend of resistance, transmissibility, treatability and other risk factors, according to international experts [41]. Although the WHO’s intention was not to prioritize public health interventions, it is worth noting that all health systems should consider investing in bat surveillance activities in bat colonies. These activities can reduce potential epidemic outbreaks due to the high incidence and mortality caused by bacteria included in these lists. The CDC estimated that more than 2.8 million people acquired a serious healthcare-associated infection due to the bacteria included in both lists, and at least 35,000 people died as a consequence of these infections [47].

### 3.3. Bias in Current Bat Microbiome Research

**Figure 4:**
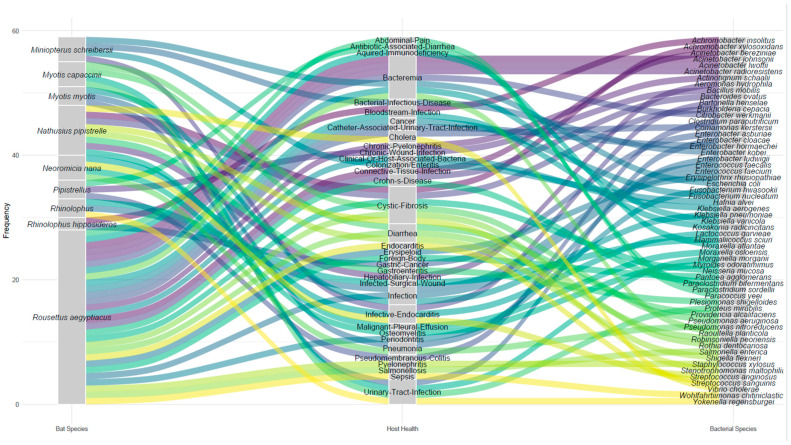
Interactions among bat species, bacterial species, and associated diseases reported in human patients from whom the bacteria were isolated in the Bacterial and Viral Bioinformatics Resource Center (BV-BRC). The colored streams represent the frequency of each connection: the thicker the line is, the greater the number of records connecting a bat species to a specific bacterium and, in turn, to a documented human disease.

Numerous studies describing the diversity of bat microbiomes have been published, but the results are generally vast and unstandardized, which is a weakness in this field. Given the amount of information generated by HTS, the data generated must follow a format for recovery and use through structured query languages by different investigators across the world. Several studies do not specify the host bat species; Figure 4 includes only those cases in which the species was explicitly identified in the analyzed literature. The lack of standardization in reporting metagenomic studies on bats introduces reporting bias and obscures possible patterns of microbial coexistence. Furthermore, the limited availability of detailed data from published manuscripts constrains the analysis of the ecological and epidemiological distributions of bacterial taxa across bat species, highlighting a significant gap in current research. Notably, *Rousettus aegyptiacus* (Linnaeus, 1758) has the highest number of reported associations with bacteria and associated diseases; however, this observation may be influenced by biases previously discussed. These findings underscore the need to standardize methodologies and reporting practices in metagenomic studies of the bat microbiome, enabling systematic integration and comparative analyses in future research.

**Figure 5:**
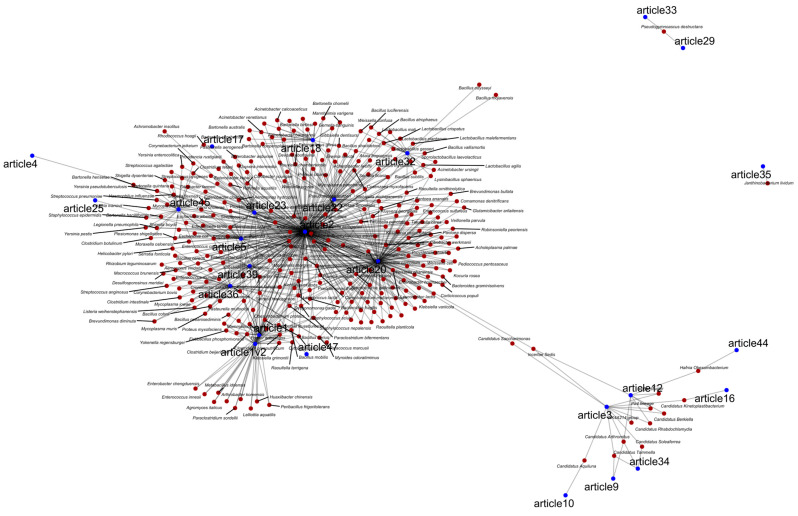
Network of articles and bacterial species shared among them. The blue dots represent articles that mention each bacterial species, the red dots indicate shared bacterial species, and the gray lines represent the connections between the articles.

Shared bacterial species are often identified as hubs in the literature (Figure 5). However, it remains unclear whether these findings represent genuine shared clinical interest or instead reflect a sampling bias toward pathogens of particular relevance. This bias may stem from the construction of reference databases commonly used for bacterial identification through bioinformatic pipelines. As a result, the observed patterns likely reflect what has been studied and disseminated rather than the true clinical prevalence of these bacteria.

Nevertheless, several bacterial species have been consistently reported across multiple studies, suggesting the presence of a conserved bacterial core within bats (see Supplementary Table S3). This observation is notable despite the need for more in-depth and targeted approaches aimed at identifying potential health indicators, which could focus on specific bacterial taxa.

Notably, *Morganella morganii* (Winslow et al., 1919) Fulton 1943, *Lactococcus garvieae* (Collins et al., 1984) Schleifer et al., 1986, *Citrobacter freundii* Werkman and Gillen, 1932, *Enterococcus faecalis* (Orla-Jensen 1919) Schleifer & Kilpper-Bälz 1984, *Klebsiella oxytoca* K. oxytoca; (Flügge 1886); Lautrop 1956, *Escherichia coli* (Migula 1895); Castellani & Chalmers 1919, and *Serratia marcescens* Bizio 1823, are among the most frequently shared species (reported in ≥8 studies) and may therefore serve as candidate health indicators, depending on their relative abundance and contextual clinical relevance.

### 3.4. High-Throughput Sequencing Approaches in Bat Microbiome Studies

To investigate the composition and dynamics of the microbiome in chiropters, HTS methods have been widely used. However, metagenomics—enabled by the ease of DNA extraction, preservation, and sequencing—is the most suitable approach over culturomics. Metagenomics is increasingly used in biosurveillance, public health, and clinical applications [59]. It plays a critical role in tracking molecular changes in pathogens that drive pandemics, aiding in refining mathematical models to assess transmission risk, geographic spread, infection dynamics, and epidemic potential [60,61].

The metagenomic workflow used in the different articles included DNA extraction, library preparation, and sequencing via short-read platforms (e.g., Illumina, Roche 454, Ion Torrent) or long-read platforms (e.g., PacBio, Oxford Nanopore) [62]. The most common HTS method used within the last five years is amplicon sequencing (targeted gene markers). This approach employs primers designed to target hypervariable regions flanked by conserved sequences within genes (16S rRNA, 18S rRNA, and ITS), which are ideal for phylogenetic identification of taxa in a sample [63]. This method provides a broad overview of community composition but offers relatively low taxonomic resolution [60,63].

For shotgun metagenomics, sequencing has typically been performed on platforms such as Illumina MiSeq, HiSeq 2500, or NovaSeq 6000, using paired-end 150- to 250-base reads (PE150–PE250). This sequencing methodology enables deeper analysis by capturing the total DNA in a sample, improving resolution and gene detection [60,63]. The accepted sequencing data output for each sample ranges from 6–9 giga-bases (GB) among the bioinformatic community. However, per-sample costs are significantly higher than those of targeted amplicon sequencing, and biases in this approach are less well characterized than those in amplicon methods. Shotgun sequencing profiles entire genomes, including those of viruses, bacteria, archaea, and eukaryotes, allowing genetic profiling through the assembly of short DNA reads, but in our review, we found that it was less preferred by the community [63]. This reflects the predominance of studies focused on taxonomy and leaves out the possibility of studying metabolic functions, resistance genes, and virulence factors or studies that delve into the genomes of the microbiota in bats.

## 4. Conclusions and Future Directions

The list of bacterial species identified in the microbiomes of various bat species is extensive and continues to expand with ongoing research. While many of these taxa are commonly found in the gastrointestinal microbiota of mammals or in natural environments, a significant proportion include genera known to be pathogenic to humans or act as opportunistic pathogens, particularly in immunocompromised individuals. Among the genera most frequently reported across multiple studies are *Mycoplasma*, *Staphylococcus*, *Pseudomonas*, *Acinetobacter*, *Enterobacter*, *Clostridium*, and *Chlamydia*. Several of these bacterial genera and species have been recognized as priority pathogens by global health organizations such as the World Health Organization (WHO) and the Centers for Disease Control and Prevention (CDC). Given the clinical severity of diseases associated with some of these bacteria, bats should be considered relevant hosts of potential zoonotic agents that warrant inclusion in current epidemiological surveillance systems. Frequently shared bacteria could serve as candidate health indicators, depending on their relative abundance (e.g., *Morganella morganii*, *Lactococcus garvieae*, *Citrobacter freundii*, *Enterococcus faecalis*, *Klebsiella oxytoca*, *Escherichia coli*, and *Serratia marcescens*). Importantly, while microbiome data from bat studies are increasingly available, the lack of standardized reporting practices limits their utility for comprehensive analyses of ecological or epidemiological patterns within bacterial communities.

Despite growing interest in bat microbiomes, the field faces major methodological inconsistencies that hinder comparisons across studies. Future research would benefit from the standardization of sample collection (e.g., clear differentiation between guano, rectal swabs, and intestinal tissues), sequencing approaches (marker gene versus shotgun metagenomics), and bioinformatic pipelines for taxonomic assignment. Adopting common metadata reporting standards would greatly enhance the reproducibility and integration of microbiome datasets across species and regions.

Beyond the zoonotic perspective, understanding how environmental disturbances, physiological stress, and anthropogenic factors shape the bat microbiome is crucial for improving animal welfare and conservation. The microbiome can reflect physiological states such as immune competence, nutritional stress, or exposure to pollutants; thus, it may serve as a valuable biomarker for assessing bat health in managed or disturbed habitats. Future lines of research should move toward functional and longitudinal studies to uncover the ecological and physiological roles of microbial communities, including their potential involvement in immunomodulation, detoxification, and pathogen resistance. Integrating microbiome data with ecological, behavioral, and health parameters will provide a more holistic understanding of the bat–microbe relationship and its implications for both wildlife conservation and public health.

## Figures and Tables

**Table 1:** Human infectious diseases caused by pathogenic bacteria found in bats.

Bacteria	Disease *
*Corynebacterium jeikeium* Jackman et al., 1988	Bacteremia, endocarditis, infections in immunocompromised patients
*Enterococcus faecalis* (Andrewes and Horder 1906) Schleifer and Kilpper-Bälz 1984	Urinary tract infections, endocarditis, sepsis
*Enterococcus faecium* (Orla-Jensen 1919) Schleifer and Kilpper-Bälz 1984	Resistant nosocomial infections (VRE)
*Moraxella osloensis* Bøvre and Henriksen 1967 (Approved Lists 1980)	Opportunistic infections (otitis, bacteremia)
*Staphylococcus saprophyticus* (Fairbrother 1940) Shaw et al. 1951 (Approved Lists 1980)	Urinary tract infection (common in young women)
*Staphylococcus lugdunensis* Freney et al., 1988	Skin infections, endocarditis
*Streptococcus suis* Hasegawa et al., 2024	Meningitis, sepsis (zoonosis from pigs)
*Campylobacter jejuni* (Jones et al., 1931) Steele and Owen 1988	Gastroenteritis, Guillain-Barré syndrome
Pasteurella canis Mutters et al., 1985	Infections from dog bites
*Chromobacterium haemolyticum* Han et al., 2008	Rare systemic infections, sepsis
*Elizabethkingia anophelis* (Kämpfer et al., 2011) García-López et al., 2020	Nosocomial infections, neonatal meningitis
*Ralstonia pickeettii* (Ralston et al., 1973) Yabuuchi et al., 1996	Opportunistic nosocomial infections
*Myroides odoratimimus* (Vancanneyt et al., 1996) García-López et al., 2020	Wound infections, urinary tract infections, pneumonia
*Aggregatibacter actinomycetemcomitans* (Klinger 1912) Nørskov-Lauritsen and Kilian 2006	Aggressive periodontitis, endocarditis
*Haemophilus parainfluenzae* Rivers 1922 (Approved Lists 1980)	Opportunistic respiratory infections
*Veillonella parvula* (Veillon and Zuber 1898) Prévot 1933 (Approved Lists 1980)	Endocarditis, polymicrobial infections
*Bacteroides fragilis* (Veillon and Zuber 1898) Castellani and Chalmers 1919 (Approved Lists 1980)	Intra-abdominal abscesses, bacteremia
*Propionibacterium acnes* McDowell et al., 2016	Acne, prosthetic device infections
*Fusobacterium nucleatum* Gharbia and Shah 1992	Periodontitis, abscesses, associated with colorectal cancer
*Parabacteroides distasonis* (Eggerth and Gagnon 1933) Sakamoto and Benno 2006	Intestinal and opportunistic infections
*Treponema denticola* (ex Flügge 1886) Chan et al., 1993	Advanced periodontal disease
*Klebsiella pneumoniae* (Schroeter 1886) Trevisan 1887 (Approved Lists 1980)	Pneumonia (including classical lobar pneumonia), urinary tract infections, sepsis, liver abscesses
*Acinetobacter baumannii* Bouvet and Grimont 1986	Nosocomial pneumonia, wound infections, sepsis, meningitis (especially in ICU)
*Staphylococcus aureus* Rosenbach 1884 (Approved Lists 1980)	Skin infections, osteomyelitis, endocarditis, pneumonia, food poisoning, sepsis
*Streptococcus pyogenes* Rosenbach 1884 (Approved Lists 1980)	Pharyngitis, scarlet fever, rheumatic fever, necrotizing fasciitis, impetigo, toxic shock syndrome
*Streptococcus agalactiae* Lehmann and Neumann 1896 (Approved Lists 1980)	Neonatal sepsis and meningitis, urinary tract infections, chorioamnionitis in pregnant women
*Streptococcus pneumoniae* (Klein 1884) Chester 1901 (Approved Lists 1980)	Neumonía, meningitis, otitis media, sinusitis, bacteriemia
*Moraxella catarrhalis* (Frosch and Kolle 1896) Henriksen and Bøvre 1968 (Approved Lists 1980)	Otitis media, sinusitis, respiratory infections (bronchitis, exacerbated COPD)
*Escherichia coli* (Migula 1895) Castellani and Chalmers 1919 (Approved Lists 1980)	Urinary tract infections, gastroenteritis (EHEC, ETEC), sepsis, neonatal meningitis
*Yersinia enterocolitica* (Schleifstein and Coleman 1939) Neubauer et al., 2000	Gastroenteritis, pseudoapendicitis, adenitis mesentérica
*Proteus mirabilis* Hauser 1885 (Approved Lists 1980)	Urinary tract infections, kidney stone formation (due to urease), bacteremia
*Providencia stuartii* (Buttiaux et al., 1954) Ewing 1962 (Approved Lists 1980)	Catheter-associated urinary tract infections, nosocomial infections in immunocompromised patients
*Serratia marcescens* Bizio 1823 (Approved Lists 1980)	Nosocomial infections: respiratory, urinary, sepsis, wound infections
*Pseudomonas aeruginosa* (Schroeter 1872) Migula 1900 (Approved Lists 1980)	Pulmonary infections (cystic fibrosis, ICU), burns, external otitis, ocular infections
*Clostridioides difficile* (Hall and O’Toole 1935) Lawson et al., 2016	Pseudomembranous colitis (after antibiotics), antibiotic-associated diarrhea, toxic megacolon
*Mycoplasma pneumoniae* Somerson et al., 1963	Atypical pneumonia, bronchitis, pharyngitis, extrapulmonary complications (rash, hemolytic anemia)
*Stenotrophomonas maltophilia* (Hugh 1981 ex Hugh and Ryschenkow 1961) Palleroni and Bradbury 1993	Respiratory, urinary tract, and bloodstream infections (mainly in immunocompromised patients)
*Aeromonas hydrophila* (Chester 1901) Stanier 1943 (Approved Lists 1980)	Diarrhea, wound infections (especially in freshwater), sepsis in immunosuppressed
*Vibrio vulnificus* (Reichelt et al., 1979) Farmer 1980	Wound infections (necrotizing), fulminant septicemia from shellfish consumption, gastroenteritis
*Pasteurella multocida* (Lehmann and Neumann 1899) Rosenbusch and Merchant 1939 (Approved Lists 1980)	Cellulitis and animal bite infections (dogs/cats), chronic respiratory infections
*Bartonella henselae* (Regnery et al., 1992) Brenner et al., 1993	Cat scratch disease (lymphadenopathy), bacillary angiomatosis (in immunocompromised)

* Reference: [44].

## Data Availability

All the data used in the analysis is within it and the script utilized to recover the information in this review can be recovered from https://github.com/jdjuliosoto/Bacterial_Composition_2020-2025 (accessed on 25 September 2025).

## Supplementary Materials

The following supporting information can be downloaded at https://www.mdpi.com/article/10.3390/ani15213126/s1. Supplementary Table S1: Metadata [42,45,64,65,66,67,68,69,70,71,72,73,74,75,76,77,78,79,80,81,82,83,84,85,86,87,88,89,90,91,92,93,94,95,96,97,98,99,100,101,102,103,104,105,106,107,108]. Supplementary Table S2: Pres_Reference. Supplementary Table S3: Shared. Supplementary Table S4: Names. Supplementary Table S5: Aerosol transmission.
